# Chrysin Attenuates Fructose-Induced Nonalcoholic Fatty Liver in Rats via Antioxidant and Anti-Inflammatory Effects: The Role of Angiotensin-Converting Enzyme 2/Angiotensin (1-7)/Mas Receptor Axis

**DOI:** 10.1155/2022/9479456

**Published:** 2022-06-08

**Authors:** Hala Attia, Norah Albekairi, Layal Albdeirat, Arwa Soliman, Reem Rajab, Hend Alotaibi, Rehab Ali, Amira Badr

**Affiliations:** ^1^Department of Pharmacology and Toxicology, College of Pharmacy, King Saud University, Riyadh 11495, Saudi Arabia; ^2^Department of Biochemistry, College of Pharmacy, Mansoura University, Mansoura 35516, Egypt; ^3^College of Pharmacy, King Saud University, Riyadh 11495, Saudi Arabia; ^4^Department of Pharmacology and Toxicology, College of Pharmacy, Ain Shams University, Heliopolis, Cairo, Egypt

## Abstract

**Aim:**

Nonalcoholic fatty liver disease (NAFLD) is the hepatic manifestation of metabolic syndrome, and if untreated, it may propagate into end-stage liver disease. The classical arm of the renin-angiotensin system (RAS) has a fundamental role in triggering oxidative stress and inflammation, which play potential roles in the pathogenesis of NAFLD. However, the nonclassical alternative axis of RAS, angiotensin- (Ang-) converting enzyme 2 (ACE2)/Ang (1-7)/Mas receptor, opposes the actions of the classical arm, mitigates the metabolic dysfunction, and improves hepatic lipid metabolism rendering it a promising protective target against NAFLD. The current study is aimed at investigating the impact of chrysin, a well-known antioxidant flavonoid, on this defensive RAS axis in NAFLD.

**Methods:**

Rats were randomly distributed and treated daily for eight weeks as follows: the normal control, chrysin control (50 mg/kg, p.o), NAFLD group (received 20% fructose in drinking water), and treated groups (25 and 50 mg/kg chrysin given orally and concomitantly with fructose). Diminazene aceturate (DIZE) (15 mg/kg, s.c.) was used as a reference ACE2 activator. *Key Findings*. High fructose induced significant weight gain, hepatocyte degeneration with fat accumulation, and inflammatory cell infiltration (as examined by H&E staining). This was accompanied by a substantial increase in liver enzymes, glucose, circulating and hepatic triglycerides, lipid peroxides, inflammatory cytokines, and Ang II (the main component of classical RAS). At the same time, protein levels of ACE2, Ang (1-7), and Mas receptors were markedly reduced. Chrysin (25 and 50 mg/kg) significantly ameliorated these abnormalities, with a prominent effect of the dose of 50 mg/kg over DIZE and the lower dose in improving ACE2, Ang (1-7), and Mas. *Significance*. Chrysin is a promising efficient protective remedy against NAFLD; mechanisms include the activation of ACE2/Ang (1-7)/Mas axis.

## 1. Introduction

Nonalcoholic fatty liver disease (NAFLD) is considered the hepatic component of metabolic syndrome [1]. The hallmark of NAFLD is the accumulation of triglycerides (TG) by more than 5-10% of liver weight, which occurs without associated secondary causes such as excessive alcohol consumption, viral or autoimmune hepatitis, or congenital hepatic disorders [2]. NAFLD is one of the main contributors to morbidity and mortality worldwide because of the rapid progression into end-stage liver disease and liver malignancy [3]. The estimated global prevalence of NAFLD is 25%, increasing to 55.5% in patients with type II diabetes mellitus (T2DM) and 60–80% in people with obesity [4, 5].

Multiple aetiologies of NAFLD have been suggested, including oxidative stress, induction of mitochondrial dysfunction, disturbance of endoplasmic reticulum, and insulin resistance (IR) [6]. In adipose tissue, IR impairs the antilipolytic action of insulin, with a resultant increase of free fatty acids (FFA) release with high circulating FFA available for subsequent hepatic uptake. In response to systemic IR, hyperinsulinemia develops, which stimulates hepatic *de novo* lipogenesis accompanied with the impairment of FFA *β*-oxidation, culminating in the accumulation of TG in the liver and the progression to fatty liver [3, 7].

The renin-angiotensin system (RAS) has a fundamental role in triggering oxidative stress and inflammation as well as regulating insulin sensitivity that is closely related to NAFLD [8], and thus, RAS was documented as one of the contributors to the development and the progression of NAFLD [9, 10]. The RAS includes a classical axis, angiotensin-converting enzyme (ACE)/angiotensin (Ang) II/type 1 angiotensin receptor (AT1R), and an alternative axis: (ACE2)/Ang 1-7/Mas receptor axis [11]. The classical RAS axis starts with the cleavage of angiotensinogen, produced in the liver, to Ang I by renin, produced by the kidney. This is followed by the ACE-catalysed conversion of Ang I to Ang II, which can bind to the AT1R [12], leading to Ang II-mediated prooxidation, inflammation, and vascular effects. This axis can contribute to the pathogenesis of NAFLD via the stimulation of IR, *de novo* lipogenesis, mitochondrial dysfunction, reactive oxygen species (ROS) generation, and production of proinflammatory cytokine [10, 12, 13].

Meanwhile, an alternative axis, ACE2/Ang (1-7)/Mas receptor, appears to function against the ACE/Ang II/AT1 axis in the liver [14–17]. Ang II is degraded by the ACE2 enzyme into Ang (1-7), which antagonizes the deleterious effects of Ang II mainly via Mas receptors [16, 18, 19]. Researchers have found that activating the ACE2/Ang1-7/Mas axis mitigates the metabolic dysfunction and prevents NAFLD [17, 18] mainly via improving hepatic IR and FFA oxidation along with inhibiting liver lipogenesis and inflammation [20–22]. Cao et al. [20] reported that, in mice, Ang (1–7)/ACE2 alleviated steatosis, oxidative stress, and inflammation induced by FFA, but deletion of ACE2 exacerbated their development. Moreover, the overexpression of ACE2 in db/db mice improved fatty liver, suggesting its potential role in preventing and treating hepatic lipid metabolism [20]. Yang et al. [18] determined that ACE2 knockouts could exacerbate high fructose-induced fat deposition in the liver, promoting inflammatory mediators such as nuclear factor kappa B (NF-*κ*B). Based on these findings, targeting the ACE2/Ang (1-7)/Mas axis could represent a pharmacological approach for protecting against NAFLD.

Several drugs are being tested for their ability to protect against or prevent NAFLD including those targeting the hepatic TG accumulation, oxidative stress, inflammation, or liver fibrosis [23], while little attention is gained toward the protective arm of RAS.

Flavonoids, which are found in plants in high concentrations, have been gaining attention in the past decade, particularly in relation to NAFLD [24]. Chrysin (5,7-dihydroxyflavone) is a well-known flavonoid found in blue passion flower (*Passiflora caerulea*), Indian trumpet flower (*Oroxylum indicum*), honey, and propolis [25]. In addition to anti-inflammatory and antioxidant properties, it inhibits atherogenesis and hyperlipidemia [25–27]. It has many pharmacological activities such as neuroprotective, antidiabetic, anticancer, nephroprotective, cardioprotective, antiarthritic, and antiasthmatic [25]. Chrysin has shown hepatoprotective effects against several hepatotoxins like ethanol [28], carbon tetrachloride [29], ammonia [30], and paracetamol [31]. Recently, chrysin has been found to reduce plasma Ang II level and to regulate the classical arm of RAS in L-NAME hypertensive rats [32]. Notably, it has been reported to ameliorate NAFLD via its antioxidant effect [33]. However, its impact on the RAS, particularly the protective arm, in NAFLD has not been studied yet. Therefore, the present work is aimed at investigating the role of chrysin in ameliorating NAFLD via the activation of the ACE2/Ang 1-7/Mas axis. The impact of chrysin on this axis was compared with diminazene aceturate (DIZE), a well-known ACE2 activator commonly used in many animal models [34–36].

Fructose (fruit sugar) is a monosaccharide present in many plants such as sugar cane, sugar beets, and corn. It is mainly added as a sweetener in the form of high-fructose corn syrup to a variety of processed foods and beverages, such as desserts, pastries, and soft drinks [37]. It is now generally thought that increased fructose consumption is one of the major causes of chronic metabolic diseases, including obesity, diabetes, and NAFLD [38, 39]. When added to highly consumed beverages, fructose can cause steatosis in only seven days [40]. Being a highly lipogenic monosaccharide, high fructose consumption leads to IR and finally fatty liver [38]. In this regard, fructose overload in chow or drinking water is a commonly used model of NAFLD in rats [41] and was used in the current study to investigate the impact of chrysin on the ACE2/Ang (1-7)/Mas axis in NAFLD.

## 2. Materials and Methods

### 2.1. Chemicals and Kits

Chrysin, DIZE, and fructose were purchased from Sigma-Aldrich (St. Louis, MO, USA). A variety of colorimetric kits were obtained from BioDiagnostic Company (Egypt) for the assay of lipid peroxides, reduced glutathione (GSH), liver enzymes, and serum levels of glucose and TG. The colorimetric kit used to quantify hepatic TG (# MAK266) was obtained from Sigma-Aldrich (St. Louis, MO, USA). ELISA kits for the assay of inflammatory markers including tumor necrosis factor-*α* (TNF-*α*), interleukin-6 (IL-6), and NF-*κ*B were provided from MyBioSource (San Diego, CA, USA). Rabbit monoclonal anti-ACE2 antibody (ab108252) was purchased from Abcam (Cambridge, MA, USA). Rabbit polyclonal anti-Mas receptor (sc135063) antibody was purchased from Santa Cruz Biotechnology. Rabbit polyclonal ant-Ang II (MBS286234) and anti-Ang 1-7 (MBS2112534) antibodies were obtained from MyBioSource (San Diego, CA, USA). Monoclonal rabbit anti-beta-actin (*β*-actin) and secondary goat anti-rabbit horseradish peroxidase- (HRP-) conjugated antibody (# 7074) were purchased from Cell Signaling Technology (Beverly, MA, USA).

### 2.2. Animals

Forty-eight Wistar rats weighed between 150 and 200 g were provided from Prince Naïf Bin Abdulaziz Health Research Center, King Saud University, Riyadh, Saudi Arabia. The animal protocol was designed to minimize pain or discomfort to the animals. The rats were placed in individual cages as four rats per cage at a temperature of 22°C ± 2°C and relative humidity of 50% ± 5% with a 12 h light/dark cycle. In addition to standard rodent chow, the rats were allowed free access to water. Before experiments began, the animals were allowed to acclimate for 1 week to the animal house conditions. The local Ethics Committee at King Saud University approved the experimental procedures (Ethics reference No: SE-19-108).

### 2.3. Study Design

The rats were weighed, randomly divided into six groups (eight rats/each), and treated daily for eight weeks as follows: Group 1: normal control received carboxymethylcellulose (CMC, 0.5% in normal saline) by oral gavage. Group 2: drug control received 50 mg/kg chrysin (dissolved in 0.5% CMC solution) by oral gavage. Group 3 was the model group given 20% fructose in drinking water [42–46] + equivalent volume of 0.5% CMC (the vehicle of chrysin) orally. Groups 4 and 5 were treated, respectively, with 25 and 50 mg/kg chrysin dissolved in 5% CMC, concomitant with 20% fructose. Group 6: rats received DIZE (15 mg/kg, s.c., dissolved in normal saline) + equivalent volume of 0.5% CMC concomitant with 20% fructose. DIZE was used as a reference ACE2 activator.

### 2.4. Preparation of Serum and Liver Homogenate

At the end of the experiment, fasted rats were reweighed and the body gain was calculated. Rats were then anesthetized with carbon dioxide, and the serum was separated from the blood after it had been collected, allowed to clot, and centrifuged for 20 minutes at 3500 rpm.

Liver tissues were removed, rinsed with normal saline, and weighed for the calculation of liver index as follows: liver index = liver weight/body weight × 100. The liver was then divided into three parts: the first part was homogenized in phosphate-buffered saline (PBS) and used for assessing hepatic TG, oxidative stress, and inflammatory markers. Another part of the liver was stored in liquid nitrogen for western blot analysis of ACE2, Ang (1-7), Mas R, and Ang II. To detect the structural changes and fat deposition, a third part was fixed in 4% formalin for histological examination using hematoxylin and eosin staining (H&E).

### 2.5. Histological Examination

A small part of liver tissue was fixed in 4% phosphate-buffered formalin at 4°C for 24 hours. After fixation, samples were dehydrated using ascending grades of alcohol, embedded in paraffin, and processed using rotary microtome to prepare 5 *μ*m thick paraffin sections. For staining, sections were deparaffinized by the incubation in a 60°C-heated oven for 1 hour, followed by the immersion in xylene for 10 minutes, and then rehydrated using descending concentrations of ethanol followed by the immersion in xylene for 10 minutes and then rehydrated using descending concentrations of ethanol. Finally, the sections were stained with H&E for examination of any structural abnormalities and inflammatory cell infiltration. The examination of the slides was performed under a light microscope, and digital images were captured using Olympus CKX 41 microscope (Olympus Optical Co., Ltd., Tokyo).

### 2.6. Assessment of Fasting Glucose, TG, and Liver Function Tests

Using the commercial kits, serum was used to assess glucose, TG, and liver enzymes including alanine aminotransferase (ALT) and aspartate aminotransferase (AST) according to the manufacturers' instructions.

### 2.7. Assay of Hepatic TG

Hepatic TAG was quantified using a colorimetric quantification kit according to the manufacturer's instructions. First, 100 mg of liver tissue was homogenized in 1 ml of 5% Nonidet P 40 (NP-40, # 74385) in distilled H_2_O. The homogenate was heated at 80–100°C in a water bath for 2–5 minutes or until the Nonidet P 40 became cloudy. The homogenate was allowed to cool to room temperature. The heating process was repeated one more time to solubilize all TG. The homogenate was then centrifuged for 2 min to remove any insoluble substances, and the supernatant was diluted 10-fold with water before the assay. Briefly, 50 *μ*l of the Master Reaction Mix was added to 50 *μ*l of each sample and standard, mixed well, and incubated for 40 minutes at room temperature. The absorbance of the colored product was measured at 570 nm.

### 2.8. Assay of Lipid Peroxidation and GSH

Hepatic levels of malondialdehyde (MDA, a final product of the peroxidation process of cell membrane lipids) and GSH (a critical nonenzymatic antioxidant) were determined using colorimetric kits according to manufacturers' instructions. For determination of MDA, 200 *μ*l of sample homogenate or standard was mixed with 1 ml of thiobarbituric acid (TBA) reagent. The mixture was incubated at a temperature of 95°C for 30 min, and the absorbance of the pink-colored TBA reactive product was measured at 535 nm against the reagent blank. For GSH, 0.5 ml of hepatic homogenate was mixed with 0.5 ml of trichloroacetic acid to precipitate protein, then the mixture was centrifuged, and 0.5 ml of the supernatant was mixed with 100 *μ*l of 5,5′-dithiobis (2-nitrobenzoic acid) reagent and the absorbance of the yellow-colored product was measured at 405 nm against reagent blank.

### 2.9. Assay of Inflammatory Markers

According to the manufacturers' instructions, the hepatic levels of the proinflammatory cytokines, TNF-*α* and IL-6, and their regulatory transcription factor, NF-*κ*B, were assayed using the ELISA technique.

### 2.10. Western Blot Analysis of Ang II, ACE2, Ang (1-7), and Mas Receptor

Ang II, ACE2, Ang (1-7), and Mas receptor protein levels were determined by Western blotting. A total of about 100 mg of liver tissue was homogenized in a phosphatase inhibitor cocktail and protease inhibitor-containing radioimmunoprecipitation assay (RIPA) buffer at ice-cold temperature. The homogenate was centrifuged, and the lysate protein concentrations were determined using a Direct Detect® infrared spectrometer (Millipore). Using polyacrylamide gel electrophoresis with sodium dodecyl sulphate as the stationary phase, the extracted proteins were separated and electrophoretically transferred to polyvinylidene difluoride membranes. After being blocked with 5% bovine serum albumin in Tris-buffered saline (TBS) at room temperature for 1 h, the blots were incubated overnight at 4°C with the primary antibodies including anti-Ang II (1 : 200), anti-ACE2 (1 : 500), anti-Ang (1-7) (1 : 1000), and anti-Mas receptor (1 : 1000). After washing with TBS, the membranes were incubated with goat anti-rabbit HRP-conjugated secondary antibodies (1 : 1000), for 2 hours at room temperature. Bio-Rad Universal Hood II Gel Doc System was used to visualize the immunoreactive bands developed using chemiluminescent detection reagents. We quantified the intensities of the different protein bands using ImageJ software and normalized them to the loading control (*β*-actin).

### 2.11. Statistical Analysis

Data were expressed as the mean ± SEM. Statistical comparisons were performed using one-way analysis of variance (ANOVA) followed by the Tukey-Kramer test as post hoc multiple tests. GraphPad Prism Software Inc. (San Diego, CA, USA) was used for the statistical analysis. Results were considered significant at a *P* value less than 0.05.

## 3. Results

### 3.1. Effect of Chrysin on Liver Architecture in High-Fructose-Induced NAFLD in Rats (Figure 1)

Liver sections from both standard control (a, b) and chrysin control (c, d) showed normal liver architecture including intact hepatic lobules with normal hepatocytes, portal areas, and central veins. However, liver sections from rats received 20% fructose alone showed loss of hepatic architecture as revealed by degeneration and ballooning of hepatocytes, pyknotic nuclei, and dilated blood sinusoids in addition to extensive fat droplet deposition (steatosis) and inflammatory cell infiltration (e, f, and g). Concomitant treatment with chrysin at dose 25 (h, i) and 50 mg/kg (j, k) showed marked improvement of hepatic architecture with remarkable attenuation of the cell degeneration, fat disposition, and inflammatory infiltration.

### 3.2. Effect of Chrysin on the Activities of Liver Enzymes (ALT, AST) in High-Fructose-Induced NAFLD in Rats (Figure 2)

In the present study, fructose feeding resulted in a significant increase in the serum levels of ALT (75.46 ± 1.84 vs. 64.45 ± 1.43 U/l, *P* < 0.01) and AST (87.46 ± 2.52 vs. 71.84 ± 2.55 U/l, *P* < 0.01) when compared to the normal control group. The fructose-induced elevation of ALT was significantly ameliorated by the treatment with 25 mg/kg (69.44 ± 1.88 vs. 75.46 ± 1.84, *P* < 0.05) and 50 mg/kg (65.65 ± 2.24 vs. 75.46 ± 1.84, *P* < 0.01) of chrysin compared to the activities in the fructose feeding group. Similarly, the high activities of AST were alleviated by the concomitant treatment with 25 and 50 mg/kg chrysin (77.9 ± 1.92 and 77.09 ± 1.9, respectively, vs. 87.46 ± 2.52, *P* < 0.05). No significant differences in liver enzyme activities were observed between the normal control and chrysin control. Also, no significant difference was observed between rats treated with 25 and 50 mg/kg chrysin.

### 3.3. Effect of Chrysin on the Weight Gain and Liver Index in High-Fructose-Induced NAFLD in Rats (Figure 3)

Rats fed with high fructose showed a highly significant weight gain compared to the normal control (86.14 ± 6.57 vs. 44 ± 6.76, *P* < 0.001). This weight gain was significantly ameliorated by the simultaneous ingestion of 25 mg/kg chrysin (59.86 ± 6.24 vs. 86.14 ± 6.57, *P* < 0.05) and to a greater extent with the dose of 50 mg/kg (48 ± 4.18 vs. 86.14 ± 6.57, *P* < 0.001) compared to the gain experienced with the fructose alone.

Concerning liver index (as a measure of fat accumulation), rats received fructose showed a significant elevation in liver index compared to the normal control (3.1 ± 0.19 vs. 2.64 ± 0.03, *P* < 0.001). However, this increase was significantly attenuated by the supplementation with 25 and 50 mg/kg of chrysin (2.73 ± 0.066 and 2.78 ± 0.07, respectively, vs. 3.1 ± 0.19, *P* < 0.001). However, no significant difference was observed between rats treated with 25 and 50 mg/kg chrysin in terms of weight gain and liver index.

### 3.4. Effect of Chrysin on the Serum and Hepatic Levels of Triglycerides (TG) in High-Fructose-Induced NAFLD in Rats (Figure 4)

**Figure 4:**
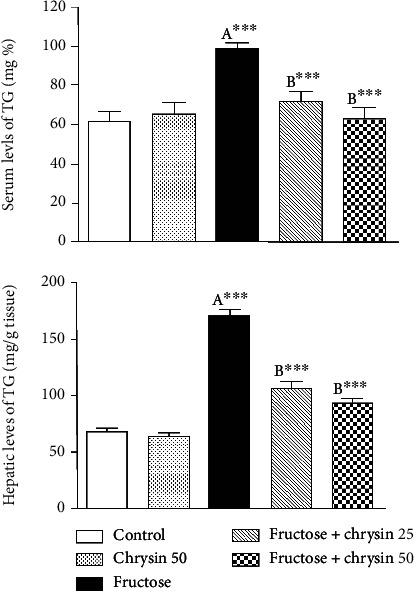
The effect of 25 and 50 mg/kg chrysin on serum and hepatic levels of triglycerides (TG) in high-fructose-induced NAFLD. Values are expressed as the mean ± SEM. (A) Significantly different from the normal control group. (B) Significantly different from fructose-induced NAFLD. ^∗∗∗^*P* < 0.001.

As revealed by Figure 4, rats received 20% fructose showed a highly significant increase in both sera (98.2 ± 2.7 vs. 61.34 ± 5.2 mg/dl, *P* < 0.001) and hepatic levels of TG (169.4 ± 5.9 vs. 67.6 ± 2.8, *P* < 0.001) compared to the normal control. Chrysin at both doses markedly improved TG levels both in serum (71.65 ± 4.5 and 62.8 ± 5.7 vs. 98.2 ± 2.7, *P* < 0.001) and liver tissue (105.6 ± 5.7 and 92.8 ± 3.5 vs. 169.4 ± 5.9, *P* < 0.001).

### 3.5. Effect of Chrysin on Serum Levels of Fasting Glucose in High-Fructose-Induced NAFLD in Rats (Figure 5)

Rats fed with 20% fructose alone revealed a marked increase in the serum fasting glucose (193.4 ± 14.99 vs. 90.08 ± 8.7 mg/dl, *P* < 0.001) compared to the normal control. Concomitant treatment with 25 and 50 mg/kg chrysin significantly alleviated the hyperglycemia induced by fructose (142.8 ± 11.3 vs. 193.4 ± 14.99, *P* < 0.05 and 125.3 ± 8.86 vs. 193.4 ± 14.99, *P* < 0.001, respectively) with a prominent effect with the dose 50 mg/kg. However, no significant difference was observed between rats treated with 25 and 50 mg/kg chrysin.

### 3.6. Effect of Chrysin on the Extent of Lipid Peroxidation and the Levels of GSH in High-Fructose-Induced NAFLD in Rats (Figure 6)

In the present study, there was a marked elevation in the hepatic levels of MDA in rats ingested with 20% fructose compared to the normal control (72.81 ± 2.54 vs. 45.32 ± 1.74 nmol/g tissue, *P* < 0.001). This elevation of MDA was associated with a significant reduction of GSH (9.6 ± 0.64 vs. 13.09 ± 0.34 mg/g tissue, *P* < 0.001), which reflects the oxidant/antioxidant imbalance in the liver. MDA elevation was alleviated by the concomitant treatment with 25 and 50 mg/kg of chrysin (57.42 ± 2.6 and 55.46 ± 2.35, respectively, vs. 72.81 ± 2.54, *P* < 0.001). In addition, the GSH depletion was attenuated by administration of 25 mg/kg (12.18 ± 0.35 vs. 9.6 ± 0.64, *P* < 0.01) and 50 mg/kg chrysin (13.06 ± 0.24 vs. 9.6 ± 0.64, *P* < 0.001).

### 3.7. Effect of Chrysin on the Hepatic Levels of Inflammatory Markers in High-Fructose-Induced NAFLD in Rats (Figure 7)

**Figure 7:**
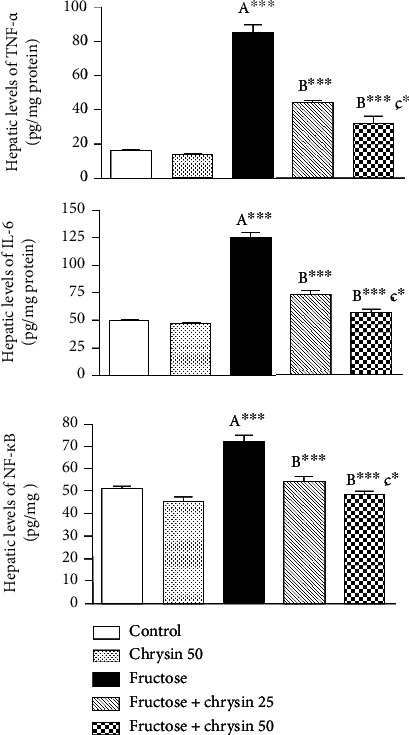
The effect of 25 and 50 mg/kg chrysin on hepatic levels of inflammatory markers, including tumor necrosis factor-*α* (TNF-*α*), interleukin-6 (IL-6), and nuclear factor kappa B (NF-*κ*B) in high-fructose-induced NAFLD. Values are expressed as the mean ± SEM. (A) Significantly different from the normal control group. (B) Significantly different from fructose-induced NAFLD. (C) Significantly different from fructose+chrysin 25. ^∗∗∗^*P* < 0.001, ^∗∗^*P* < 0.01, and ^∗^*P* < 0.05.

As shown in Figure 7, there are dramatic increases in the hepatic levels of inflammatory markers including TNF-*α* (84.2 ± 5.2 vs. 15.8 ± 0.6 pg/mg protein, *P* < 0.001), IL-6 (123.9 ± 4.6 vs. 48.1 ± 2.02 pg/mg protein, *P* < 0.001) and NF-*κ*B (72.3 ± 3.2 vs. 45.3 ± 1.7 pg/mg tissue, *P* < 0.001) in rats received 20% fructose compared to the normal control. This inflammatory response was markedly ameliorated by chrysin at 25 and 50 mg/kg (*P* < 0.001). The dose 50 mg/kg revealed significant improvement in TNF-*α* (31.1 ± 4.6 vs. 43.8 ± 1.7, *P* < 0.05), IL-6 (56.2 ± 3.6 vs. 72.4 ± 3.9, *P* < 0.05) and NF-*κ*B (48 ± 1.4 vs. 55 ± 2.1, *P* < 0.05) compared to the levels elicited by the dose 25 mg/kg.

### 3.8. Effect of Chrysin on the Protein Levels of Ang II in High-Fructose-Induced NAFLD in Rats (Figure 8)

In the present study, a high intake of fructose led to an approximately 6-fold increase in protein levels of Ang II (*P* < 0.001), reflecting stimulation of the classical axis of RAS as an essential mechanism of the NAFLD induced by fructose. Cotreatment with both 25 and 50 mg/kg of chrysin significantly mitigated the elevation of Ang II (3.16 ± 0.3 and 2.1 ± 0.11-fold, respectively, vs. 5.75 ± 0.31-fold, *P* < 0.001) with the prominent effect of the dose 50 mg/kg compared to the dose 25 mg/kg (*P* < 0.01). It is noted that the dose 50 mg/kg elicited significant mitigated the expression of Ang II compared to DIZE (2.1 ± 0.11 vs. 3.18 ± 0.22, *P* < 0.01).

### 3.9. Effect of Chrysin on the Protein Levels of ACE2, Ang (1-7), and Mas Receptors in High-Fructose-Induced NAFLD in Rats (Figure 9)

Our results revealed that high fructose caused dramatic depletion of protein levels of ACE 2, Ang (1-7), and Mas receptor by approximately 70% (*P* < 0.001) compared to the normal control supporting that the impairment of this RAS axis is implicated in fructose-induced NAFLD. The protein levels of ACE 2, Ang (1-7), and Mas receptors were significantly improved by the treatment with DIZE, chrysin 25 (*P* < 0.01), and to a greater extent with chrysin 50 mg/kg (*P* < 0.001). Chrysin (50 mg/kg) has a notable superior effect over DIZE and chrysin (25 mg/kg) in improving the levels of ACE-2 (*P* < 0.05), Ang (1-7) (*P* < 0.001), and Mas receptor (*P* < 0.05).

## 4. Discussion

The main finding of the present study is that chrysin at doses 25 and 50 mg/kg alleviates the protein expression of Ang II in the liver while enhancing ACE2, Ang (1–7), and Mas receptor protein expression in the fructose model of NAFLD. The effect of the lower dose is comparable to the impact of DIZE, while the higher dose elicits a superior impact than the DIZE and the lower dose. Therefore, chrysin may contribute to reducing the progression of NAFLD via modulating the hepatic RAS.

The high-fructose model of NAFLD in experimental animals has been widely used as a reliable and reproducible one [41]. In this regard and consistent with previous works [46–48], fructose in the present study was associated with liver injury associated with increased fat accumulation, as documented by elevated hepatic TG content and fat droplets observed after H&E staining.

In the presence of liver damage, the cytosolic enzymes of hepatocytes, ALT and AST, are released into the bloodstream [49]. This study found that fructose feeding significantly raised the levels of ALT and AST in serum, suggesting the increased permeability of the cell membrane that triggers the leakage of these intracellular transaminases into circulation. This result is consistent with previous works [33, 38, 39] and may be explained in terms of high-fructose-induced formation of ROS, which attack phospholipids in membranes, causing cell membrane rupture [33, 38]. Chrysin at either dose (25 or 50 mg/kg) significantly inhibited the serum elevation of liver enzymes, reflecting the membrane stability induced by chrysin. This result coincided with a remarkable attenuation of the cell degeneration in H&E staining. The effect of chrysin to on liver function marker enzymes is previously reported [33, 50–54] and could be explained in terms of its antioxidant characteristics [25].

In the liver, TG is derived from dietary sources or *de novo* lipogenesis. The consumption of high-fructose diets is documented to be lipogenic [38, 47]. This is attributed to its rapid uptake and utilization through the liver. Inside the liver, fructose is rapidly metabolized by fructokinase to fructose-1-phophate that plays a role as a carbon atom donor for the synthesis of TG [48]; thus, fructose can be considered as an efficient inducer of *de novo* lipogenesis and increased secretion and hepatic storage of TG [48]. In addition, dietary fructose increases *de novo* lipogenesis by upregulating the lipogenic enzymes that accelerate TG synthesis in the hepatocytes [40, 55]. Fat accumulates in the liver as a result of this enhanced lipogenesis is associated with increased liver weight and body weight [33, 56] which could explain the significant weight gain and liver index observed in our study in the rats fed with high fructose. The results of these experiments are consistent with our observations where the serum and hepatic TG levels increased significantly with fructose consumption simultaneously with fat deposition and fat infiltration, as observed by H&E staining. The weight gain, high liver index, and elevated TG levels (both in the serum and liver tissue) observed in the current study were markedly reduced by chrysin supplementation. These data are in agreement with those of Pai et al. [33] and Pushpavalli et al. [57] and support the findings concerning the antihyperlipidemic effects of chrysin [26, 27, 58–60], which is exerted probably via elevating the activity of lipoprotein lipase (LPL) which hydrolyses the extra TG [27] and downregulating the action of the lipogenic enzymes, e.g., fatty acid synthase [59].

Fructose also induced hyperglycemia. This is because the liver converts a significant amount of fructose into glucose [55]. Moreover, the high levels of TG in the bloodstream, caused by fructose, cause IR, thereby adds to increasing blood sugar levels [48]. These findings are in harmony with our results and the work of Tappy [55] and Pai et al. [33]. Both doses of chrysin significantly alleviated this hyperglycemia, indicating the ability of chrysin to reduce IR and improve glucose metabolism in fructose-induced NAFLD; this result is consistent with recent work by Pai et al. [33] and Satyanarayana et al. [61].

In the pathogenesis of high-fructose-induced NAFLD, oxidative stress plays a crucial role [46, 47]. An indication of oxidative stress in this study is the augmented hepatic levels of MDA which is associated with depleted levels of GSH, an essential cellular antioxidant that acts against redox imbalance restoring insulin sensitivity in obesity-associated metabolic syndrome [62]. These findings reflect the oxidant/antioxidant imbalance in the liver, which results in oxidative stress. Here, we reported that chrysin (25 and 50 mg/kg) significantly ameliorated this imbalance, suggesting increased scavenging ability of free radicals and the hepatoprotective effect via antioxidant properties. The chrysin's antioxidant activity has been reported in several animal models via different mechanisms [50–53, 60, 63–65].

Due to the generation of ROS triggered by high fructose in addition to fat accumulation, hepatic proinflammatory cytokines will be continuously generated from the Kupffer cells [3, 66]. There is evidence that NAFLD is associated with elevated TNF-*α* and IL-6 levels, cytokines that play a crucial role in systemic and local inflammation [67]. In this context, our results also showed significantly higher levels of TNF-*α* and IL-6 after fructose feeding than when fed with a standard control diet. It is also reported that oxidative stress will trigger inflammatory pathways involving NF-*κ*B [3]. It has been demonstrated that NF-*κ*B contributes to the development of NAFLD and steatohepatitis by regulating the expression of several genes involved in the inflammatory process [68–70]. The highly expressed levels of NF-*κ*B in the current study could explain the inflammatory response evoked in our model of NAFLD. The fructose-induced inflammatory response was markedly ameliorated by chrysin, particularly with the dose of 50 mg/kg. Similarly, several studies demonstrated the hepatoprotective activity of chrysin by reducing the production of inflammatory cytokines [33, 50, 52, 63, 64]. This effect could be attributed to the suppression of NF-*κ*B. In many other studies, NF-*κ*B was a common pathway by which chrysin has been shown to exert its beneficial effects [52, 64, 65, 71].

In the next step, we investigated whether the protective role of chrysin against NAFLD has mediated via the modulation of RAS, either the harmful classical axis (represented by Ang II) or the alternative protective arm comprising ACE2/Ang 1-7/Mas receptor. The effects of chrysin were compared with those elicited by DIZE, a well-known ACE2 activator.

Prior studies demonstrated that the hepatic local classical RAS axis is upregulated during NAFLD [18, 72, 73]. RAS's classical arm is dominated by Ang II which contributes to NAFLD pathogenesis through multiple mechanisms, including the induction of IR, *de novo* lipogenesis, mitochondrial dysfunction, ROS generation, and proinflammatory cytokine production [10, 12]. Ang II-infused rats, for example, displayed increased IL-6 expression in the liver, as well as increased monocyte recruitment and overall inflammation [74]. In addition, elevated Ang II is associated with increased FFAs, resulting in FFA flux to the liver and promoting the increase of TG [75]. Frantz et al. [17], in a study of rats with high fructose intake, found that ACE activity and Ang II protein expression increased which is consistent with our own finding, indicating that the classical RAS axis may play a role in the pathogenesis of high-fructose-induced NAFLD. This augmentation of Ang II may contribute to oxidative stress, inflammation, hyperglycemia, and hypertriglyceridemia induced by fructose. Cotreatment with both doses of chrysin, particularly 50 mg/kg, significantly mitigated the elevation of Ang II, suggesting an association between the hepatoprotective effect mediated by chrysin and the normalization of the classic axis of RAS. The prior study revealed that chrysin administration led to a reduction in plasma Ang-II levels in comparison to rats untreated. Free radical scavenging activity and increased plasma NO activity might explain this effect, which regulates the RAS system and reduces plasma Ang-II levels [32].

An important role of ACE2 in the RAS involves its role in degrading Ang II in order to produce cytoprotective effects. ACE2 shows its protective effects by reducing Ang II and producing Ang (1–7) [76, 77], which, by binding to Mas receptors, antagonizes the detrimental effects of Ang II [16, 18, 19]. Studies have shown that Ang (1–7) can improve glucose tolerance, insulin sensitivity, glucose uptake, and TG and cholesterol levels, accompanied with reduced abdominal fat mass [15]. Accordingly, there has been evidence that the activation of the ACE2/Ang1-7/Mas receptor axis protects against NAFLD through multiple mechanisms including inhibition of hepatic lipogenesis, enhancement of FFA oxidation, and inhibition of inflammation [20–22]. Cao et al. [20] showed that the deletion of ACE2 enhanced hepatic steatosis, oxidative stress, and inflammation in ACE2 knockout mice, and that both proteins, ACE2 and Ang (1–7), ameliorated inflammation, oxidative stress, and hepatic steatosis in FFA-induced HepG2 cells. Further, they demonstrated that overexpression of ACE2 reduced hyperglycemia and fatty liver in db/db mice. They proposed that Ang (1-7)/ACE2/Mas pathway effect is mediated through regulation of lipid-metabolizing genes [20]. According to Yang et al. [18], mice with a high-fructose diet gained significantly more body weight and liver weight than the controls, and that the lack of ACE2 further contributed to this effect. This suggests that enhancing hepatic ACE2/Ang (1-7)/MAS may provide a therapeutic strategy for counteracting the detrimental effects of high fructose in liver tissue.

In the current study, we reported that high fructose led to a dramatic decrease in the protein levels of ACE2, Ang (1-7), and Mas receptor in hepatic tissue, supporting that the impairment of this RAS axis is involved in high-fructose-induced NAFLD. Similarly, in a rat model of fructose-fed NAFLD [17], a dysregulation of the hepatic RAS coupled with an upregulation of ACE/ACE2, AngII/Ang (1-7), and Mas/AT1R ultimately led to liver steatosis. Simultaneous treatment with DIZE, chrysin 25, and chrysin 50 showed a marked improvement of the protein levels of ACE 2/Ang 1-7/Mas receptor with a prominent effect of the dose 50 mg/kg over DIZE or the lower dose of chrysin. This finding indicates that the beneficial effects, such as hypoglycemic, hypolipidemic, and anti-inflammatory effects exerted by chrysin in this model of NAFLD, could be mediated via enhancing the expression of ACE2/Ang (1-7)/Ras receptor axis of RAS in the liver. Using chrysin to modulate this RAS axis suppresses the inflammation induced by fructose ingestion, so we concluded that the ACE2/Ang (1-7)/MAS is downregulated during NAFLD and that it is essential for chrysin in preventing liver damage. Chrysin upregulates the ACE2/Ang (1-7)/Mas axis and antagonizes fatty liver. These findings provide a novel insight into the mechanism of chrysin in NAFLD therapy.

## 5. Conclusion

Daily supplementation with chrysin at either 25 or 50 mg/kg body weight can be used to protect NAFLD efficiently. Depleting the augmented levels of Ang II and upregulating the components of the protective axis of RAS, including ACE2, Ang (1-7), and Mas, represent a potential protective mechanism, particularly with the dose of 50 mg/kg.

## Figures and Tables

**Figure 1:**
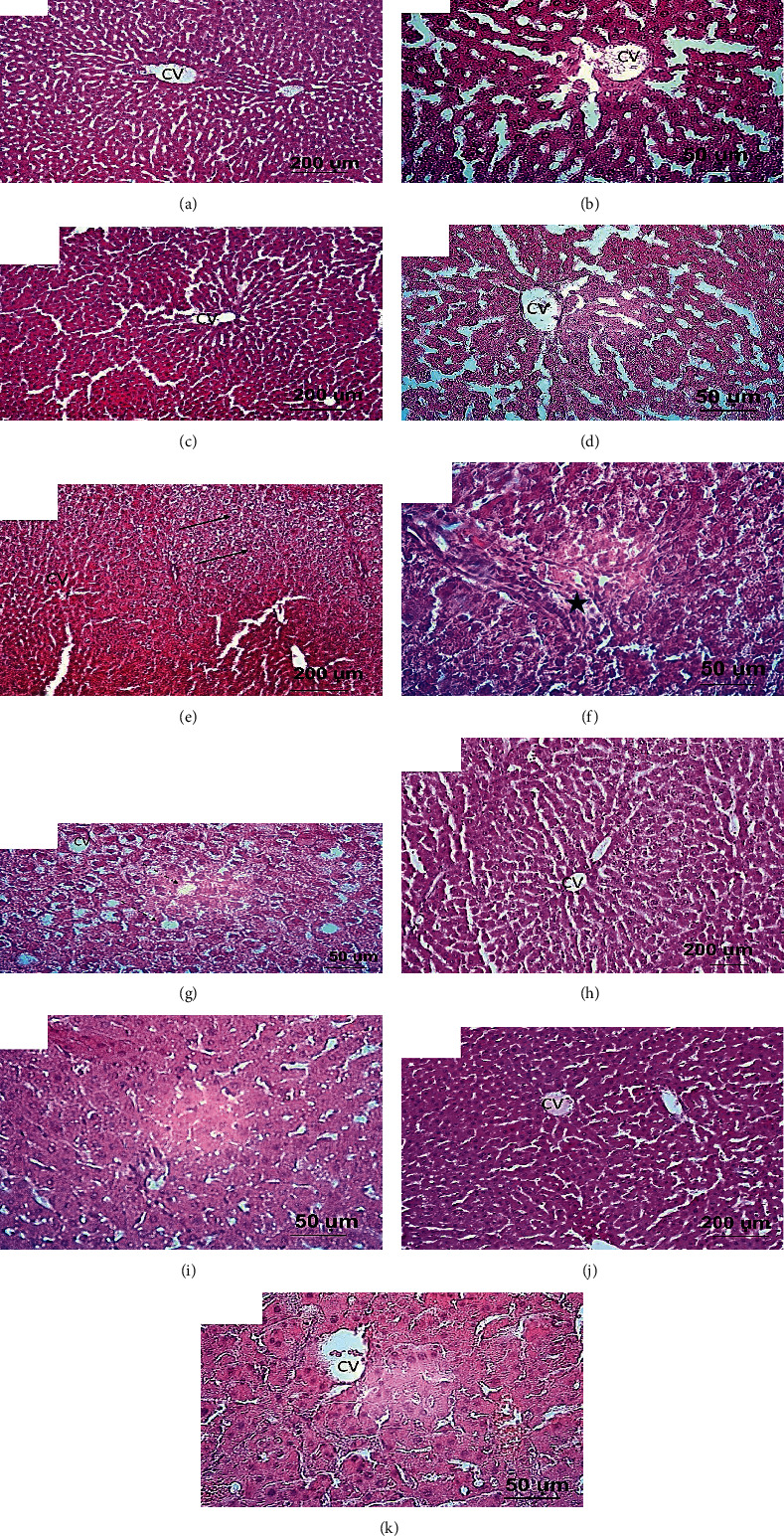
Light photographs of liver sections stained with H&E. (a, b) Liver sections from the normal rat group showing normal hepatic architecture which comprised of intact hepatic lobules with normal hepatocytes, portal area, and central veins (CV). (c, d) Liver sections from rat received only chrysin (50 mg/kg) showing apparently healthy hepatic lobules with normal hepatocytes appearance. (e, f, g) Liver sections from rats received 20% fructose showing loss of intact liver architecture which characterized by extensive fat droplet disposition (steatosis, dashed arrows), ballooning of hepatocytes and pyknotic nuclei (arrows), and inflammatory cell infiltration (star). Additionally, the blood sinusoids are dilating. (h, i) Liver sections from rat received 20% fructose and concomitantly treated with chrysin (25 mg/kg). (j, k) Liver sections from rat received 20% fructose and concomitantly treated with chrysin (50 mg/kg), showing marked improvement in the hepatic appearance with lesser or completely lacking of fatty disposition and inflammatory cells.

**Figure 2:**
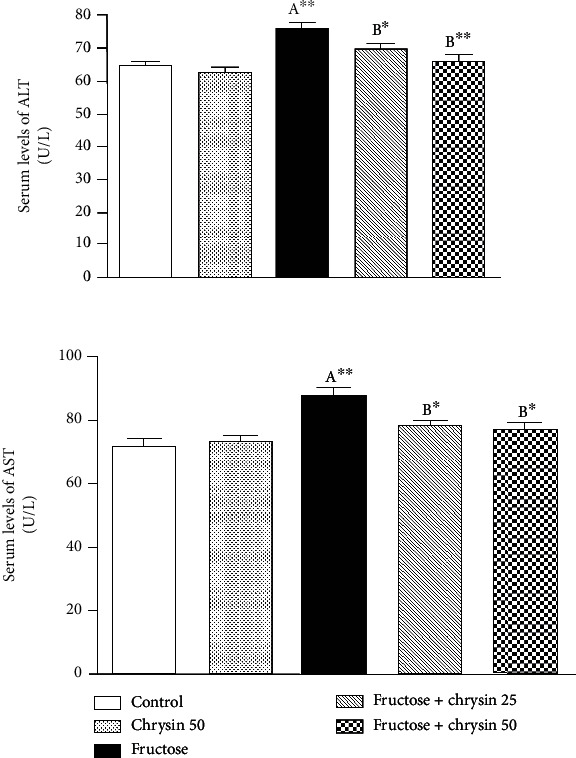
The effect of 25 and 50 mg/kg chrysin on alanine aminotransferase (ALT) and aspartate aminotransferase (AST) in high-fructose-induced NAFLD. Values are expressed as the mean ± SEM. (A) Significantly different from the normal control group. (B) Significantly different from fructose-induced NAFLD. ^∗∗^*P* < 0.01 and ^∗^*P* < 0.05.

**Figure 3:**
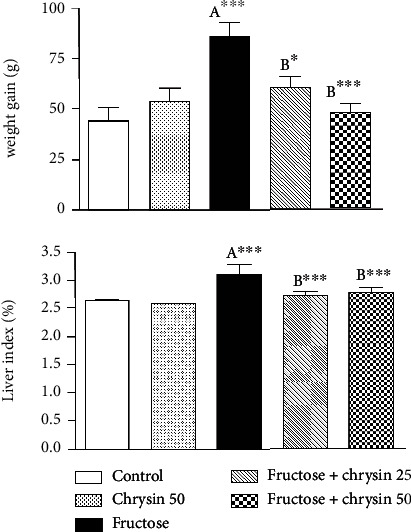
The effect of 25 and 50 mg/kg chrysin on body weight gain and liver index in high-fructose-induced NAFLD. Values are expressed as the mean ± SEM. (A) Significantly different from the normal control group. (B) Significantly different from fructose-induced NAFLD. ^∗∗∗^*P* < 0.001 and ^∗^*P* < 0.05.

**Figure 5:**
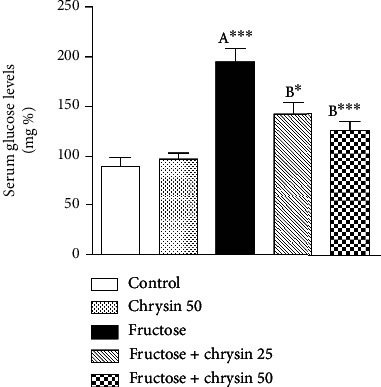
The effect of 25 and 50 mg/kg chrysin on serum levels of fasting blood glucose in high-fructose-induced NAFLD. Values are expressed as the mean ± SEM. (A) Significantly different from the normal control group. (B) Significantly different from fructose-induced NAFLD. ^∗∗∗^*P* < 0.001 and ^∗^*P* < 0.05.

**Figure 6:**
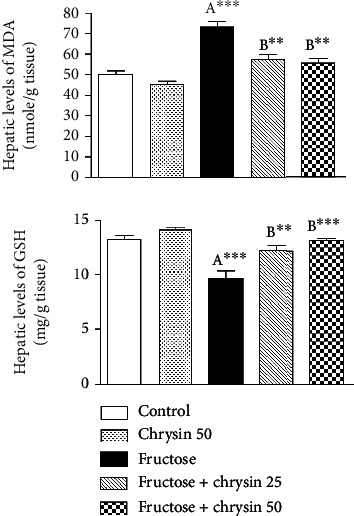
The effect of 25 and 50 mg/kg chrysin on hepatic levels of malondialdehyde (MDA) and reduced glutathione (GSH) in high-fructose-induced NAFLD. Values are expressed as the mean ± SEM. (A) Significantly different from the normal control group. (B) Significantly different from fructose-induced NAFLD. ^∗∗∗^*P* < 0.001 and ^∗∗^*P* < 0.01.

**Figure 8:**
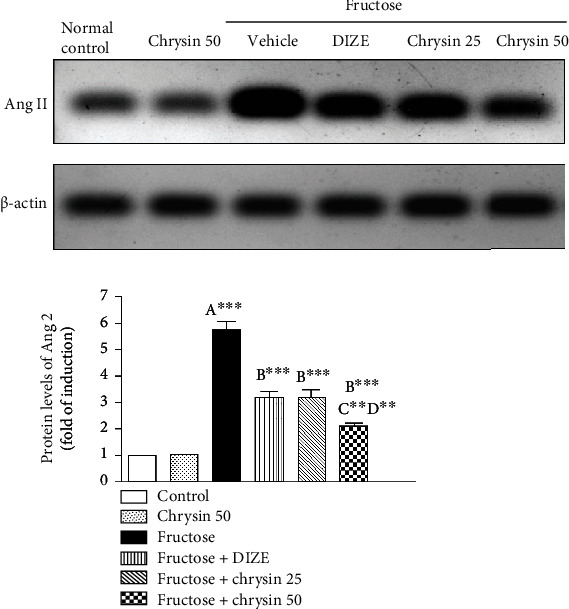
Representative immunoblots and quantitative analysis of the protein levels of Ang II in liver tissues of the controls, NAFLD, DIZE, and chrysin-treated groups. ^∗∗^*P* < 0.01 and ^∗∗∗^*P* < 0.001. (A) Significantly different from the normal control group. (B) Significantly different from fructose-induced NAFLD. (C) Significantly different from fructose+chrysin 25. (D) Significantly different from fructose+DIZE.

**Figure 9:**
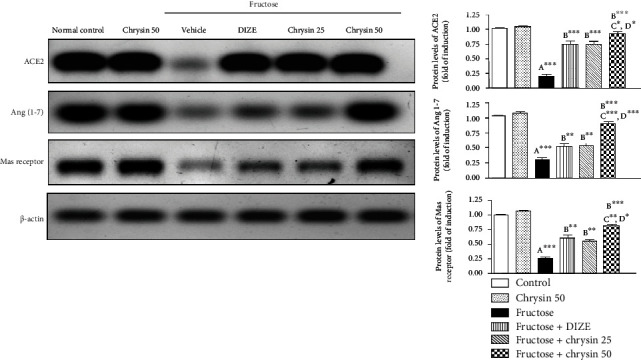
Representative immunoblots and quantitative analysis of the protein levels of ACE2, Ang 1-7, and Mas receptor in liver tissues of the controls, NAFLD, DIZE, and chrysin-treated groups. ^∗^*P* < 0.05, ^∗∗^*P* < 0.01, and ^∗∗∗^*P* < 0.001. (A) Significantly different from the normal control group. (B) Significantly different from fructose-induced NAFLD. (C) Significantly different from fructose+chrysin 25. (D) Significantly different from fructose+DIZE.

## Data Availability

The biochemical and molecular data used to support the findings of this study are included within the article.

## Abbreviations

ACE: Angiotensin-converting enzyme
ALT: Alanine aminotransferase
Ang II: Angiotensin II
AST: Aspartate aminotransferase
AT1R: Type 1 angiotensin receptor
CMC: Carboxymethylcellulose
DIZE: Diminazene aceturate
FFAs: Free fatty acids
GSH: Reduced glutathione
H&E: Hematoxylin and eosin staining
HRP: Horseradish peroxidase
IL-6: Interleukin-6
IR: Insulin resistance
MDA: Malondialdehyde
NAFLD: Nonalcoholic fatty liver disease
NF-*κ*B: Nuclear factor kappa B
PBS: Phosphate-buffered saline
RAS: Renin-angiotensin system
ROS: Reactive oxygen species
TBS: Tris-buffered saline
TG: Triglycerides
TNF-*α*: Tumor necrosis factor-*α*.
